# *Wolbachia* bacteria in *Mansonella perstans* isolates from patients infected in different geographical areas: a pilot study from the ESCMID Study Group for Clinical Parasitology

**DOI:** 10.1186/s13071-025-06723-0

**Published:** 2025-03-10

**Authors:** Elena Pomari, Denis Voronin, Miriam J. Alvarez-Martinez, Marta Arsuaga, Emmanuel Bottieau, María Pilar Luzón-García, Beatrice Nickel, Jose Miguel Rubio, Joaquín Salas-Coronas, Fernando Salvador, Manuel Jesús Soriano-Pérez, Elena Sulleiro, Lidia Goterris, Marjan Van Esbroeck, Jaap J. van Hellemond, Linda J. Wammes, Lorenzo Zammarchi, Denise Lavezzari, Monica Degani, Stefano Tais, Jana Held, Federico Gobbi, Francesca Tamarozzi

**Affiliations:** 1 Department of Infectious-Tropical Diseases and Microbiology, IRCCS Sacro Cuore Don Calabria Hospital, 37024 Negrar Di Valpolicella, Verona, Italy; 2 Systems Genomic Section, Laboratory of Parasitic Diseases, Division of Intramural Research, NIAID, NIH, Bethesda, MD 20894 USA; 3 Microbiology Department, Hospital Clinic, 08036 Barcelona, Spain; 4 ISGlobal, University of Barcelona, 08036 Barcelona, Spain; 5 National Referral Unit for Imported Tropical Diseases and Health Travel, Hospital La Paz-Carlos III, 28034 Madrid, Spain; 6 Centro de Investigación Biomédica en Red de Enfermedades Infecciosas (CIBERINFEC), Instituto de Salud Carlos III, 28029 Madrid, Spain; 7 Department of Clinical Sciences, Institute of Tropical Medicine, 2000 Antwerp, Belgium; 8 Tropical Medicine Unit, Hospital Universitario Poniente, El Ejido, 04700 Almería, Spain; 9 Swiss Tropical and Public Health Institute, 4123 Allschwil, Switzerland; 10 University of Basel, 4001 Basel, Switzerland; 11 Malaria & Emerging Parasitic Diseases Laboratory, National Centre of Microbiology, Instituto de Salud Carlos III, 28221 Madrid, Spain; 12 Department of Nursing, Physiotherapy and Medicine. Faculty of Health Sciences, University of Almeria, 04120 Almeria, Spain; 13 International Health Unit Vall d’Hebron-Drassanes, Infectious Diseases Department, Vall d’Hebron University Hospital, PROSICS Barcelona, 08035 Barcelona, Spain; 14 Microbiology Department, Vall d’Hebron University Hospital, PROSICS Barcelona, 08035 Barcelona, Spain; 15 Department of Medical Microbiology and Infectious Diseases, Erasmus MC University Medical Center, 3015 Rotterdam, The Netherlands; 16 Leiden University Center for Infectious Diseases (LUCID), Leiden University Medical Center, 2333 ZA Leiden, Netherlands; 17 Struttura Organizzativa Dipartimentale Malattie Infettive e Tropicali, Azienda Ospedaliero-Universitaria Careggi, 50134 Florence, Italy; 18 Dipartimento di Medicina Sperimentale e Clinica, Università degli Studi di Firenze, 50134 Florence, Italy; 19 Institute of Tropical Medicine, Eberhard Karls University Tuebingen, 72074 Tuebingen, Germany; 20 German Center for Infection Research, Partner Site Tübingen, 72074 Tübingen, Germany; 21 Centre de Recherches Médicales de Lambaréné, 242 Lambaréné, Gabon; 22 Department of Clinical and Experimental Sciences, University of Brescia, 25121 Brescia, Italy

**Keywords:** *Mansonella perstans*, *Wolbachia*, Filariasis, Mansonellosis, Geographical distribution, Treatment implications

## Abstract

**Background:**

*Mansonella perstans* is a vector-borne filarial parasite widely endemic in sub-Saharan Africa, with sporadic cases in Latin America. Infection is often overlooked; treatment is not standardized, and effectiveness of common regimes is difficult to ascertain. Anti-*Wolbachia* macrofilaricidal treatment with doxycycline has been applied, but there are scant and contrasting reports about the presence of *Wolbachia* in *M. perstans* isolates from different geographical locations. Taking advantage of a network of European centres expert in traveller and migrant health, we aimed to expand the knowledge concerning the distribution of *Wolbachia* in *M. perstans* to contribute to the design of optimal treatment approaches.

**Methods:**

We analysed 19 samples of concentrated microfilariae or whole blood from *M. perstans*-infected patients who reported having resided or travelled in one or more of 10 West African countries. *Wolbachia* was detected by PCR targeting 16S and ftsZ genes and phylogenetic analysis of *M. perstans* was performed based on COX1 gene sequencing.

**Results:**

*Wolbachia* was identified in 14/19 (74%) samples. With the possible inaccuracy deriving from potential origin of infection being identified retrospectively from routine clinical visit’s documents, this study identified *Wolbachia* in *M. perstans* from Burkina Faso, Equatorial Guinea, Republic of Guinea and Senegal for the first time to our knowledge. Furthermore, *Wolbachia* might also be present in *M. perstans* from Democratic Republic of the Congo, Mali, Niger and Nigeria.

**Conclusions:**

The retrieval of *Wolbachia*-positive and *Wolbachia*-negative *M. perstans* samples can either be explained by technical limitations or reflect the real existence of *Wolbachia*-positive and *Wolbachia*-negative *M. perstans* populations. However, this latter hypothesis was not supported by our phylogenetic analysis. Our results suggest that doxycycline could be used for the treatment of *M. perstans* infection upfront or, if possible, after ascertaining the presence of *Wolbachia* by PCR performed on concentrated microfilariae using two targets to avoid false-negative results.

**Graphical Abstract:**

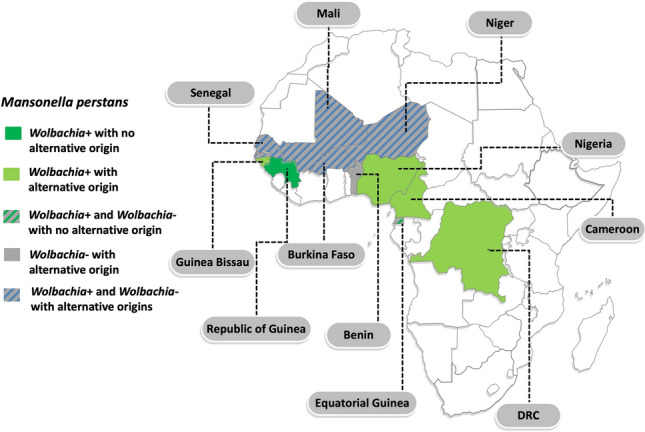

**Supplementary Information:**

The online version contains supplementary material available at 10.1186/s13071-025-06723-0.

## Background

*Mansonella perstans* is a vector-borne filarial parasite endemic in sub-Saharan Africa, with cases also occurring in the Caribbean and Central and South America [1, 2]. It is estimated that 100 million people are infected, with prevalences up to 70–100% reported in areas of Cameroon, Ghana and Uganda [1–5]. *Mansonella perstans* is also diagnosed in migrants from sub-Saharan Africa to Europe [6], with prevalences reported between 4–11% [7–10]. However, these figures are probably underestimated since infection is often overlooked because of its inconstant positivity on serology screening for filariasis and unspecific clinical presentation [6, 11–14]. Despite being considered low pathogenic, the real health impact of mansonellosis is not well defined. Treatment of *M. perstans* is not standardized and in some cases not even applied [6, 15]. Even outside endemic areas, where re-infection cannot occur, refinement of treatment schedules and assessment of macrofilaricidal activity is extremely difficult. Parasitological cure can only be indirectly evaluated by monitoring microfilaraemia or serology over months to years [16] due to the inaccessibility of adult worms living in connective tissues and serosal cavities. However, the long follow-up required to assess cure is not easily implementable in mobile populations such as migrants, and positivity at a too early follow-up might not reflect treatment failure and lead to unnecessary repeated treatments.

Prolonged courses of mebendazole, alone or in combination with diethylcarbamazine, seem the most effective treatment strategies, but their effectiveness in reaching parasitological cure is uncertain [15, 16]. Treatment with doxycycline for 4–6 weeks targeting *Wolbachia* has reliably proven macrofilaricidal on filariae harbouring *Wolbachia* endosymbionts such as *Wuchereria bancrofti* and *Onchocerca volvulus* [17]. However, there are still contrasting reports and overall scant data about the presence of *Wolbachia* in *M. perstans* isolates from different geographical locations and, therefore, the applicability upfront of doxycycline for the treatment of this infection. Early investigations from Uganda and Gabon did not find evidence of *Wolbachia* in *M. perstans* microfilariae using PCR [18, 19]. However, later studies performed in Gabon and Cameroon [20, 21] and clinical trials with doxycycline in Mali [22, 23] and Ghana [24], showed that *Wolbachia* was present in *M. perstans* and that treatment was effective in significantly reducing microfilaraemia, probably reflecting an adulticidal effect. In these studies, 40–50% [20, 23] to 75–100% [22, 24] of blood samples with *M. perstans* microfilariae were *Wolbachia*-positive. Notably, PCR for *Wolbachia* is not standardized; therefore, different results could also derive from the application of different pre-analytical and analytical procedures even when the same targets are used (e.g. 16S rDNA and ftsZ that are the most common targets used for *Wolbachia* PCR [18, 20, 23, 24]). Interestingly, Sinha et al., [21] recently showed that two different *M. perstans* isolates from Cameroon had different loads of *Wolbachia* bacteria per microfilaria, suggesting that *Wolbachia* levels can vary widely between parasite populations. To complicate the picture further, a new *Mansonella* species, *Mansonella* sp. “DEUX”, morphologically indistinguishable from *M. perstans*, was identified in Gabon [25–27]. *Wolbachia* was detected by PCR in 33% *Mansonella* sp. “DEUX” mono-infections isolates [26].

Taken together, available data suggest that *Wolbachia* is present in *M. perstans.* However, the co-existence of *Wolbachia*-free and *Wolbachia*-positive populations cannot be excluded. In this project, taking advantage of a network of European centres specialized in traveller and migrant health, we aim to expand the knowledge concerning the distribution of *Wolbachia* in *M. perstans* populations to contribute to the design of optimal treatment approaches to this neglected filarial disease.

## Methods

### Study design and objectives

In this cross-sectional study, we analysed isolated *M. perstans* microfilariae and *M. perstans*-positive samples, collected from infected patients during routine diagnostic procedures for filariasis, irrespective of whether the diagnosis was based on investigations of suggestive signs/symptoms (e.g. eosinophilia) or in the context of a geographically oriented screening (e.g. positivity on screening with filarial serology) already available or prospectively collected between April 2021-March 2024. The objectives were to evaluate the presence of *Wolbachia* in *M. perstans* microfilariae obtained from patients and the distribution of *Wolbachia*-positive and *Wolbachia*-negative *M. perstans* populations according to the geographical origin of the infection.

### Study population and data collection

Samples were obtained from patients diagnosed with *M. perstans* infection who consented to their storage and use for research purposes. Inclusion criteria were: (i) *M. perstans* infection was diagnosed by molecular analysis or identification of microfilariae using microscopy; (ii) the sample was collected before treatment with doxycycline (for retrospective samples, treatment information was derived from the medical records); (iii) the sample was processed and stored according to study requirements (see sample processing eligibility criteria in the “Molecular analysis” paragraph below); (iv) the patient’s country of birth and/or the possible country/countries of infection was available. Samples from patients with *M. perstans* infection which did not meet inclusion criteria were excluded from the study.

Other data retrieved from medical records, if available, were: patient’s sex, age, quantity of blood processed for the original diagnosis, diagnostic method (microscopy after sedimentation/filtration, molecular analysis), number of microfilariae per ml blood and co-infection with other filarial species.

### Molecular analyses

Sample processing eligibility criteria were: (i) microfilariae were retained on filters after blood filtration or obtained from leukoconcentration/sedimentation techniques routinely implemented in each centre without application of formalin or acetic acid, stored at − 80 °C or in ethanol; (ii) whole blood stored at − 80 °C; (iii) DNA extracted from whole blood or concentrated microfilariae, stored at − 80 °C. No specific requirements were applied to type of collection tube or length of sample storage.

DNA was extracted from concentrated microfilariae or 500 µl whole blood using a QIAmp Blood and Tissue DNA extraction kit (Qiagen, Hilden, Germany) following the manufacturer’s instructions. Briefly, frozen samples were thawed, mixed with lysis buffer and proteinase K, mechanically disrupted with ceramic bead-beating (MagNA Lyser, Roche, Basel, Switzerland) and incubated at 56 °C overnight. DNA was purified on columns and eluted in 50 µl AE buffer.

PCR reactions were performed using primers and TaqMan probes targeting filarial ITS1 and *Wolbachia* ftsZ and 16S (Supplementary Table S1). A pre-amplification was performed with endpoint PCR for the ITS1 gene (20 cycles) and *Wolbachia* ftsZ and 16S genes (30 cycles). The reaction mixture was composed of HotStarTaq Master Mix (Qiagen) and primers at 400 nM final concentration in a final volume of 25 µl. *Mansonella perstans*, *Mansonella* sp. “DEUX” and *Wolbachia*-specific real-time PCR reactions (45 cycles amplification) were performed. *Wolbachia*-negative *Loa loa* [19] was confirmed using specific real-time PCR (ITS1) after pre-amplification for samples with co-infection by microscopy. Real-time PCR mix was composed of SsoAdvanced Universal Probes Supermix (BioRad, Hercules, CA, USA) with primers at 400 nM and probes at 200 nM final concentrations in a final volume of 25 µl. To exclude the possibility that *Wolbachia* positivity was derived from submicroscopic infections with *W. bancrofti*, the presence of this parasite was investigated using the oligonucleotides reported in [28]; pre-amplification and real-time PCRs were performed as described above.

A first set-up of the molecular analyses was carried out using DNA extracted from control samples positive for *M. perstans, L. loa* and *W. bancrofti*. Subsequently, analyses on the study samples were performed using synthetic amplicons as positive control and water as negative (no template) control.

### Sequencing and phylogenetic analysis

All study samples (Table 1) were analysed using Sanger sequencing for the COX1 region (464 bp) using primers reported in Supplementary Table S1. PCRs were performed using HotStarTaq Master Mix (Qiagen) at 60 °C for the annealing. PCR products were purified by enzymatic method (ExoSAP-IT™ PCR Product Cleanup Reagent, Thermo Fisher Scientific, Waltham, MA, USA) followed by cycle sequencing with BigDye™ Terminator v3.1 kit (Thermo Fisher Scientific). Sequences were analysed with the ABI3500 instrument (Applied Biosystems, Thermo Fisher Scientific). Data were analysed together with COX1 genes available in NCBI Nucleotide (search query: “cox1 AND ("Mansonella perstans"[ORGN])”, acc. date: 18/10/2024), selecting only from humans. A multiple sequences alignment (MSA) was done using MAFFT v. 7.520 (2023/Mar/22) with option –maxiterate 1000. The resulting MSA was used for the phylogenetic analysis with IQ-TREE v. 2.3.5 (options: -keep-ident, -model-joint UNREST, –root-test, -B 1000, -T auto). TreeViewer v. 2.2.0 was used to visualize the tree.

**Table 1:** Origin/potential origin of *Wolbachia*-positive and *Wolbachia*-negative samples

Patient ID no.	GenBank ID	Birth country (if considered primary country of infection)	Other reported possible countries of infection	*Wolbachia* pos/neg		
				Final identification	16S	ftsZ
#1	PQ525011	Cameroon	–	POS	−	+
#2	PQ525012	–*	Cameroon, DRC	POS	−	+
#3	PQ525001	Burkina Faso	Niger	POS	+	-
#4	PQ525002	Senegal	Mali, Niger	POS	+	+
#5	PQ525003	Senegal		POS	+	−
#6	PQ525004	Equatorial Guinea		NEG	−	−
#7	PQ525005	Senegal		POS	+	+
#8	PQ525006	Senegal	Mali, Burkina Faso, Niger	NEG	−	−
#9	PQ525007	Guinea Bissau	Senegal, Mali	POS	+	+
#10	na	Senegal	Mali, Burkina Faso, Niger	NEG	−	−
#11	PQ525009	Senegal	–	POS	+	+
#12	na	–*	Cameroon	POS	+	−
#13	PQ525010	Senegal	–	NEG	−	−
#14	PQ525247	Equatorial Guinea	–	POS	+	−
#15	PQ524992	Benin	Niger	NEG	−	−
#16	PQ524993	Cameroon	–	POS	+	+
#17	na	Burkina Faso	–	POS	+	−
#18	PQ524994	Cameroon	Niger, Nigeria	POS	+	+
#19	PQ524995	Republic of Guinea	–	POS	+	+

*DRC* Democratic Republic of the Congo. * = European country of birth. *na*  not available

### Statistical analysis

Samples were considered *Wolbachia*-positive if at least one gene (*Wolbachia* ftsZ and/or 16S) was amplified. Data are described as counts and percentages. Microfilarial load (mf/ml blood) in the original sample and starting quantity of blood analysed for the diagnosis were graphically compared between *Wolbachia*-positive and *Wolbachia-*negative groups.

## Results

### Patients’ origin and other possible countries of infection

**Fig. 1:**
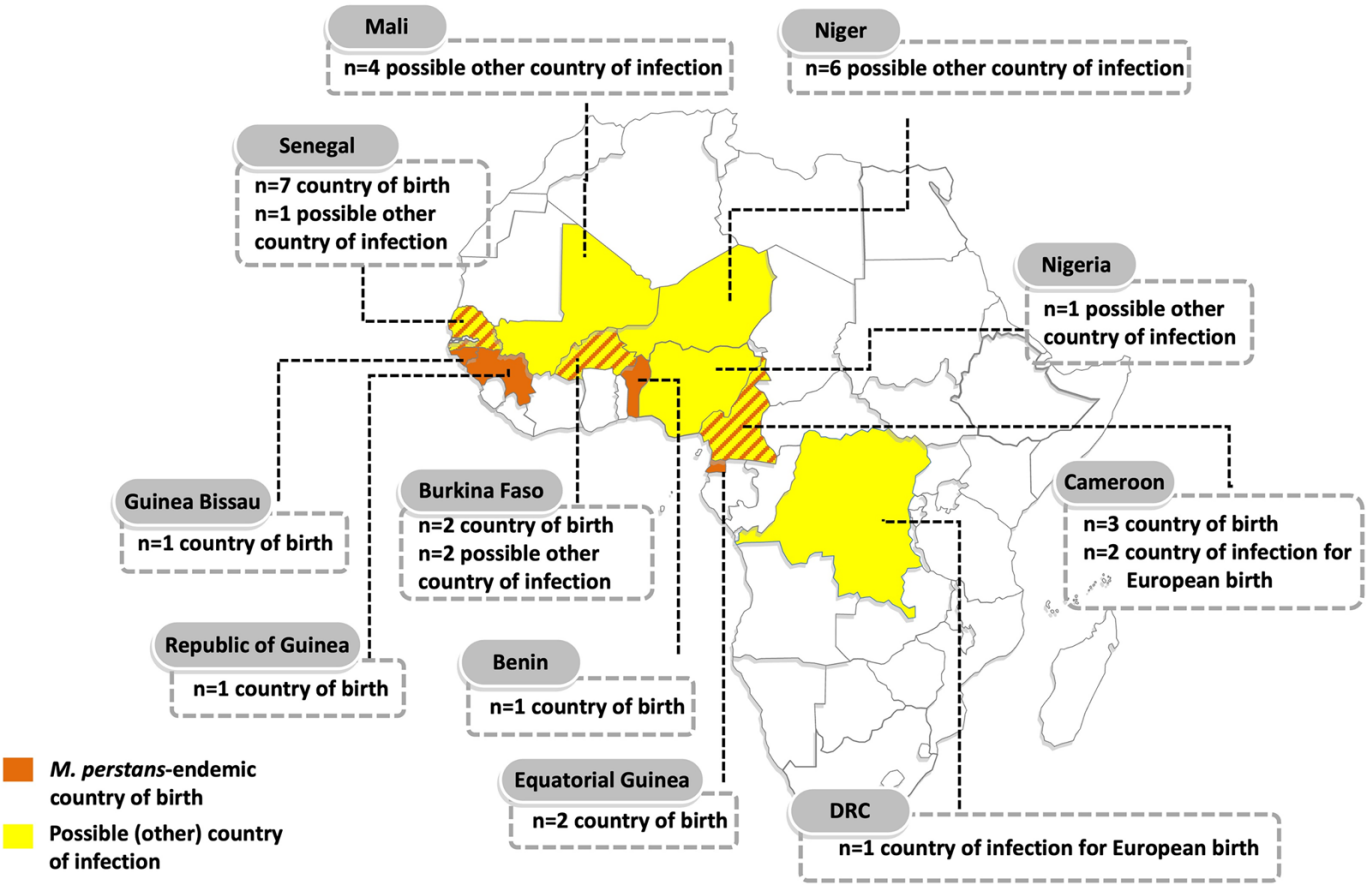
*Mansonella perstans*-endemic country of birth (orange) or other possible country of infection (yellow) of patients from whom samples were analysed. *DRC* Democratic Republic of the Congo. Map produced using www.yourfreetemplates.com

Nineteen samples were available for the analysis: 15/19 (79%) were concentrated microfilariae from 10 ml (*n* = 7) or 1 ml (*n* = 8) blood, 3/19 (16%) were frozen whole blood samples from which the original diagnosis was made by PCR, and 1/19 (5%) samples were extracted DNA from 200 µl blood originating from a sample on which the diagnosis was made by microscopy. In 14/19 (74%) cases, patients were males; the median age was 26 (range 17–69) years. Figure 1 shows patients’ country of birth (orange) and reported possible countries of infection (yellow) if more than one endemic country was visited by the patient (*n* = 9 African patients) or if the country of birth was not endemic for *M. perstans* (*n* = 1 from Belgium and *n* = 1 from Spain). In two cases, *L. loa* co-infection was diagnosed by microscopy of concentrated blood. Detailed per-patient information is available at https://zenodo.org/records/14197836.

### Filariae identification and *Wolbachia* detection

*Mansonella perstans, Mansonella sp.* “DEUX” and *L. loa* identifications were confirmed by real-time PCRs for filarial ITS1 on reference control DNA samples. The *Wolbachia* 16S gene was amplified only in *M. perstans* whilst ftsZ was amplified in *M. perstans and Mansonella* sp. “DEUX”.

Analysing the study samples, *M. perstans* infection was confirmed in all 19 samples; no *Mansonella* sp. “DEUX” was identified. *Loa loa* co-infections on microscopy were also confirmed by molecular analysis; no submicroscopic infection with *W. bancrofti* was detected.

**Fig. 2:**
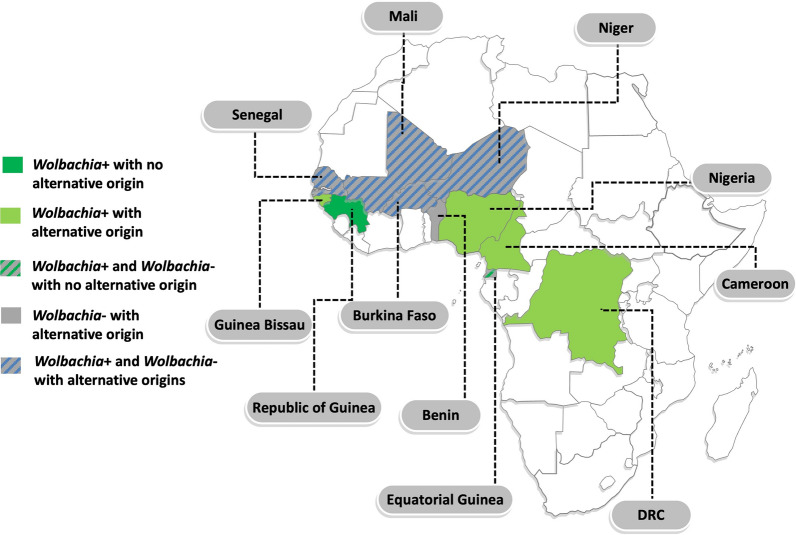
Origin or potential origin of *Wolbachia*-positive and *Wolbachia*-negative samples. Dark green: only *Wolbachia*-positive isolates and no reported alternative origin. Light green: only *Wolbachia*-positive isolates with reported alternative origin of *Wolbachia*-positive isolates. Grey: only *Wolbachia*-negative isolates with reported alternative origin of *Wolbachia*-negative isolates. Green-grey striped: both *Wolbachia*-positive ad *Wolbachia*-negative isolates with no reported alternative origin. Blue-grey striped: both *Wolbachia*-positive and *Wolbachia*-negative isolates with reported alternative origins of both positive and negative samples. *DRC* Democratic Republic of the Congo. Map produced using www.yourfreetemplates.com

*Wolbachia* was identified in 14/19 (74%) samples (Table 1). Amplification of both 16S and ftsZ genes was obtained in 7/14 (50%) specimens, while the remaining were positive only for 16S (5/14; 36% samples) or ftsZ (2/14; 14% samples). The origin or potential origin of *Wolbachia*-positive and *Wolbachia*-negative samples is detailed in Table 1 and Fig. 2.

**Fig. 3:**
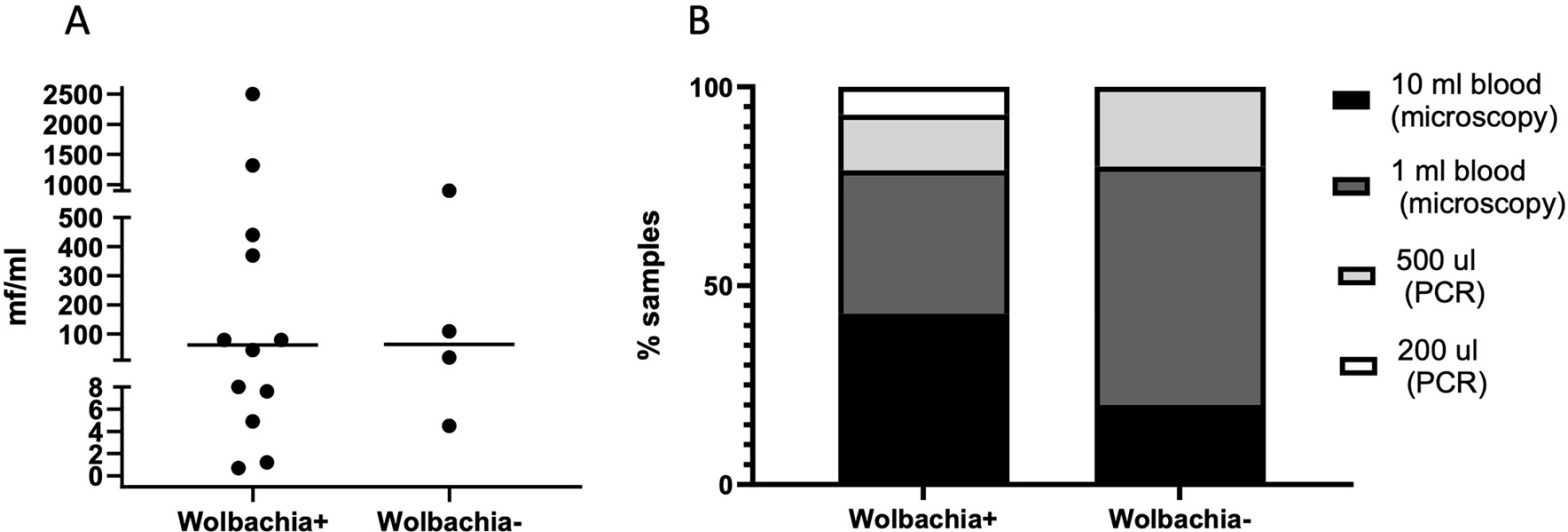
Relation between characteristics of blood samples originally used for diagnosis and positivity or negativity of *Wolbachia* PCR. **A** Original microfilarial burden (mf/ml) in *Wolbachia*-positive and *Wolbachia*-negative samples (data available for *n* = 16/19 samples). The horizontal line indicates median value. **B** Percentage of *Wolbachia*-positive and *Wolbachia*-negative samples originating from DNA extracted from concentrated microfilariae from 10 ml blood (black), from concentrated mf from 1 ml blood (dark grey) and from 200 µl (light grey) or 500 µl (white) whole blood

No evident difference between *Wolbachia*-positive and *Wolbachia*-negative samples could be observed in terms of original microfilarial burden, while the starting quantity of blood could have been relevant (Fig. 3). Statistical analysis was not performed because of the small number of samples and lack of standardization of original diagnostic procedures and thus the quantity of material available for this study.

### Phylogenetic analysis

**Fig. 4:**
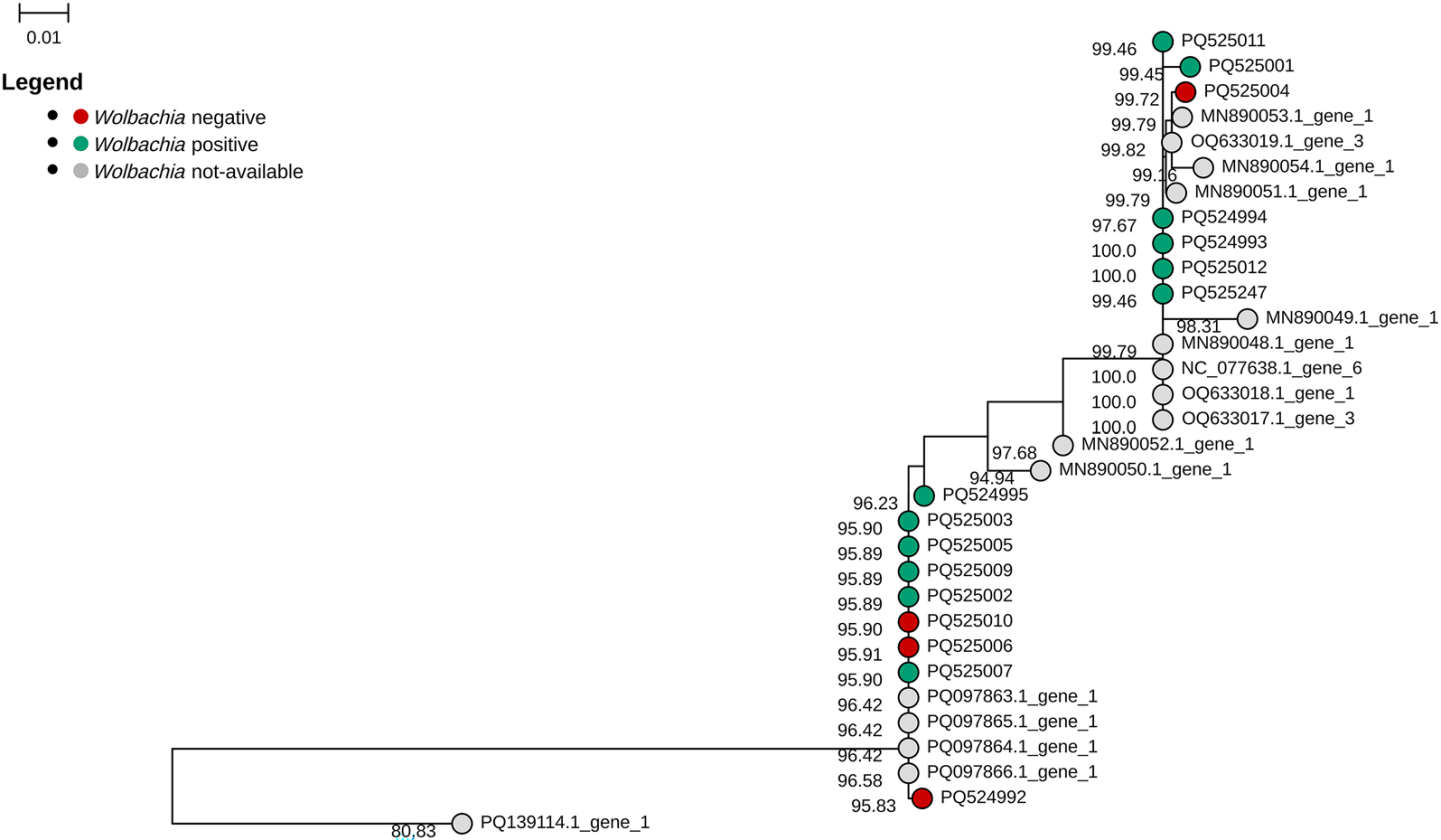
Phylogenetic analysis of *M. perstans* samples based on the mitochondrial gene COX1. The figure shows the phylogenetic analysis performed with IQ-TREE v.2.3.5 using the UNREST model with bootstrap replicates of 1000 on our 16 samples and the sequence available in NCBI database. The numbers on nodes indicate the percentage of identity with the reference genome *M. perstans* NC_077638.1. The colors on tips represent the *Wolbachia* status (red = *Wolbachia* negative, green = *Wolbachia* positive, grey = no information on *Wolbachia*)

To evaluate whether presence or absence of *Wolbachia* could relate to the presence of different *M. perstans* populations, we performed a phylogenetic analysis by MSA of the mitochondrial COX1 gene sequences obtained in this study and available in NCBI. Sixteen of 19 (84%) samples could be sequenced. *M. perstans* grouped into two branches; however, *Wolbachia*-positive and *Wolbachia*-negative samples could be found in both (Fig. 4), suggesting no separate evolution of *Wolbachia*-positive and *Wolbachia*-negative *M. perstans* populations.

## Discussion

In this study, we aimed to expand the knowledge about the presence of *Wolbachia* bacteria in *M. perstans* isolates from different geographical origins.

We confirmed the presence of *Wolbachia* in 14/19 (74%) *M. perstans* samples, suggesting that *Wolbachia* is consistently present in *M. perstans* and, for a practical clinical approach, doxycycline can be used to treat *M. perstans* infection as a first line provided that monitoring of cure is possible or after ascertaining the presence of *Wolbachia* by PCR performed on concentrated microfilariae from 10 ml blood and using two PCR targets to minimize false-negative results. No *Mansonella* sp. “DEUX” isolates were identified in our sample cohort; the distribution of this new species beyond Gabon therefore remains to be elucidated.

*Wolbachia*-positive *M. perstans* samples were obtained from patients born in Burkina Faso, Cameroon, Equatorial Guinea, Republic of Guinea, Guinea Bissau and Senegal and born in Europe but reportedly having been infected in Cameroon or DRC. Four of the African patients reported one or more possible countries of infection in addition to the country of birth, including Mali, Niger and Nigeria. Although with possible inaccuracy deriving from countries of potential infection being identified retrospectively from travel history collected during the routine clinical visit, this study identified *Wolbachia* in *M. perstans* solely attributed to infection in Burkina Faso, Equatorial Guinea, Republic of Guinea and Senegal and confirmed the presence of *Wolbachia* in isolates from Cameroon for the first time to our knowledge. Furthermore, *Wolbachia* might also be present in *M. perstans* from DRC, Mali, Niger and Nigeria. *Wolbachia* was not identified in 5/19 (26%) isolates. Equatorial Guinea and Senegal were the only reported countries of infection of two patients; the other three patients came from Senegal or Benin and reported having travelled in Burkina Faso, Mali or Niger.

The identification of both *Wolbachia*-positive and *Wolbachia*-negative *M. perstans* samples could be explained by technical limitations of the DNA amplification or reflect the real existence of *M. perstans* populations containing and free of the bacteria. When focusing on the first hypothesis, the results could originate from (i) differences in the type and quantity of sample analysed, influencing the eventual number of microfilariae in the volume used for DNA extraction, and (ii) the recently reported variability in *Wolbachia* levels among *M. perstans* populations [21]. As for the second hypothesis, the presence of *Wolbachia*-positive and *Wolbachia*-negative isolates from the same country and the findings of the phylogenetic analysis support its rejection and point in favour of the result being a technical issue rather than a real biological feature, but this could not be formally excluded because of the uncertain origin of the infections. Prospective studies applying the same pre-analytical procedures, or randomized clinical trials with doxycycline, would be more appropriate to address this hypothesis. In the absence of an animal model for *M. perstans* mansonellosis, clinical trials with doxycycline are also needed to address the symbiotic role of *Wolbachia* and its requirement for survival in *M. perstans*. Indeed, such a symbiotic role is suggested by the response to treatment in clinical trials [22–24] and has been conclusively ascertained for other filarial species [17] but still remains to be formally confirmed in *M. perstans*. However, such prospective studies are extremely difficult to perform in clinical centres attended by migrants because of the different locally available routine diagnostic procedures and capacities, the long time required to reach an adequate sample size prospectively and the difficulty in performing an adequate follow-up in this highly mobile population. Further studies in different endemic countries could overcome the sampling issue and mitigate the uncertainty deriving from possible multiple origins of infection in migrants. The recently described in vitro culture system for *M. perstans* L3 from *Culicoides* vectors [29] could also offer an interesting alternative to using microfilariae (thus, overcoming the problems deriving fromlow/variable *Wolbachia* loads per microfilaria and possible coinfection with *Wolbachia*-positive and *Wolbachia*-negative *M. perstans* parasites), allowing the analysis of pre-adult *M. perstans* stages while also avoiding human sampling.

This study has several limitations, the first being the limited number of samples available. This was due to the unspecific clinical presentation of mansonellosis, which makes its diagnosis possible de facto only in the context of screening of patients from endemic areas, or in case of strong clinical suspicion, and the uneven influx of patients in clinical centres. Furthermore, storage of samples for future studies cannot be routinely implemented in many hospitals, limiting the availability of suitable stored samples. Second, much information such as the countries where the patients resided or travelled and previous treatments received, was retrieved from visit reports and could have been inaccurate or incomplete. Furthermore, no data on length of stay (or other travel information) in each country of possible infection were reliably available. However, while missing data on the countries visited could impact the results of our study, we believe it is improbable that long-term treatment with doxycycline (or any other antibiotics that target *Wolbachia*) right before diagnosis could have been missed and therefore influenced the results of the study. Third, samples originated only from West-Central Africa. Therefore, the distribution of *Wolbachia* in *M. perstans* from other endemic areas could not be examined. Furthermore, the pre-analytical procedures of samples were not standardized. Finally, we could not formally exclude infection with the *Wolbachia*-containing *O. volvulus* in patients from which samples were derived, nor did we perform *O. volvulus*-specific PCR on the subset of whole blood samples available (the negative results of which, in any case, would not have excluded infection with certainty since *O. volvulus* microfilariae do not circulate in blood). However, we believe that this probably would not have confounded the results of our study since (i) any *Onchocerca*-suspected nodule in a patient attending a centre involved in the study would have been palpated, reported in the clinical records and further investigated; (ii) the diagnosis of onchocercosis in migrants to Europe is exceedingly rare [30]; (iii) only a few patients with ochocerciasis have detectable levels of *Wolbachia* in their blood before microfilaricidal treatment, which causes massive release of *Wolbachia* and clinical side effects [31, 32].

## Conclusions

Taken together, our results support the hypothesis that *Wolbachia* is present in *M. perstans* from West Africa. Our results also support the clinical management of patients with *M. perstans* infection with doxycycline if follow-up is possible. An alternative approach, especially for patients from East Africa and Latin America, where the presence of *Wolbachia* in *M. perstans* is unexplored, could be deciding on treatment on a case basis based on results of PCR for *Wolbachia*.

## Supplementary Information

Supplementary Material 1. Text S1. Ethical clearance from the participating centres.

Supplementary Material 2. Table S1. Primers and probes for filarial species and *Wolbachia* detection.

## Data Availability

The raw data file is available at https://zenodo.org/records/14197836. Amplicon sequences were submitted to GenBank, accession numbers PQ525011-PQ525012, PQ525001-PQ525010, PQ525247, PQ524992-PQ524995.

## Abbreviations

DRC: Democratic Republic of the Congo
ESCMID: European Society of Clinical Microbiology and Infectious Diseases
MSA: Multiple sequences alignment
