# Supplementary material for: Wolbachia bacteria in Mansonella perstans isolates from patients infected in different geographical areas: a pilot study from the ESCMID Study Group for Clinical Parasitology
Source: Parasit Vectors. 2025 Mar 10;18:97. doi: 10.1186/s13071-025-06723-0 (PMC11895188; doi:10.1186/s13071-025-06723-0)
Supplement: Supplementary file 1 — Supplementary Material 1. Text S1. Ethical clearance from the participating centres. [file 13071_2025_6723_MOESM1_ESM.docx]

**Text S1.** Ethical clearance from the participating centres.

Erasmus MC University Medical Centre Rotterdam, Rotterdam, The Netherlands: Erasmus MC MEC 2012-047; Instituto de Salud Carlos III, Madrid, Spain: CEI PI 74_2020 of 30/09/2020 and CEI PI 100_2022 of 23/01/2023; Hospital Universitari Vall d’Hebron, Barcelona, Spain: PR(AG)344/2021, 06-08-2021; Hospital Universitario Poniente, El Ejido, Almeria, Spain: Ethics Committee of Almería. Study code: RED_21_24TropNet. 21/05/2021; Azienda Ospedaliera Universitaria Careggi, Firenze, Italy: BIO_25910, 27-02-2024; Swiss Tropical and Public Health Institute, Basel, Switzerland: Ethics committee of Northwest and Central Switzerland EKNZ UBE-15/22, EKNZ Req-2026-00050; ISGlobal Hospital Clinic Barcelona, Barcelona, Spain: reception of PI’s ethics approval, independent ethic approval not required for this type of study; Leiden University Medical Center, Leiden, The Netherlands: LUMC Review Committee Biobank & biomaterials (TCBio) February 29, 2024; Institute of Tropical Medicine Antwerp, Antwerp, Belgium: Institutional Review Board number 1509/21, 18/06/2021; Hospital Universitario La Paz, Madrid, Spain: PI-4934, 23-09-2021
