# Supplementary material for: Wolbachia bacteria in Mansonella perstans isolates from patients infected in different geographical areas: a pilot study from the ESCMID Study Group for Clinical Parasitology
Source: Parasit Vectors. 2025 Mar 10;18:97. doi: 10.1186/s13071-025-06723-0 (PMC11895188; doi:10.1186/s13071-025-06723-0)
Supplement: Supplementary file 2 — Supplementary Material 2. Table S1. Primers and probes for filarial species and Wolbachia detection. [file 13071_2025_6723_MOESM2_ESM.docx]

**Table S1.** Primers and probes for filarial species and *Wolbachia* detection.

| **Target** | **Oligonucleotide** | **Sequence (5’ → 3’)** | **Amplicon length (bp)** | **Reference** |
| --- | --- | --- | --- | --- |
| 18S-5.8S | Filaria pre-amp_F  Filaria pre-amp_R | CCTGCGGAAGGATCATTAWC  TCGCACTATTTATCGCAGCTAG | ~460 | Sandri et al |
| ITS1 | Mansonella sp-ITS1-F  Mansonella sp-ITS1-R  M perstans-ITS1-probe  M DEUX-ITS1-probe | CGGAAGGATCATTAACGAGCTT  CGAATATCACCGTTAATTCAGTTGT  [HEX]GCAACATGCATGAGTATATACATAT[BHQ1]  [FAM]CTGTATGTATATAGTTGCTTTGCTATT[BHQ1] | ~190 | Sandri et al |
| ITS1 | Loa loa-ITS1-F | TGATGATGATATATGATGAAGAAAC | 150 | Sandri et al |
|  | Loa loa-ITS1-R  Loa loa-ITS1-probe | TAGCTAAAATGCTTATTAAGTCTAC  [CY55]CGCCTAACCGTCGATAACAATG[BHQ2] |  |  |
| ftsZ | pre-Wolb ftsZ F  pre-Wolb ftsZ R | ATYATGGARCATATAAARGATAG  TCRAGYAATGGATTRGATAT | 523 | Sandri et al |
| ftsZ | qWolb ftsZ F  qWolb ftsZ R  qWolb ftsZ probe | ACRGCRGGAATGGGTGGTG  TTTGYAATTCYTCAAGTCCRAG  [FAM]GTTGTAACCAAGCCATTTGGCT[BHQ1] | 199 | Sandri et al |
| 16S | q16S_wMp_F  q16S_wMp_R  q16S_wMp_probe | AAGGCTCAACTTTGGAATTGCTTTT  ACTGGTGTTCCTCCTAATATTT  [ROX]AAACTGCTAACCTAGAGATTGA[BHQ2] | 110 | Keiser et al |
| COX1 | COX1_F  COX1_R | CGTTGGTCAGCCAGAGATGT  GCCCTCAGACAGAAGTACCC | 464 | This study |
| LDR | LDR1_F  LDR2_R  LDR_probe | ATTTTGATCATCTGGGAACGTTAATA  CGACTGTCTAATCCATTCAGAGTGA  [FAM]ATCTGCCCATAGAAATAACTACGGTGGATCTCTG[BHQ1] | 90 | Rao et al |

Sandri TL, Kreidenweiss A, Cavallo S, Weber D, Juhas S, Rodi M, et al. Molecular epidemiology of

Mansonella species in Gabon. J Infect Dis. 2021;223:287–96. DOI: 10.1093/infdis/jiaa670

Keiser PB, Coulibaly Y, Kubofcik J, Diallo AA, Klion AD, Traoré SF, et al. Molecular 31 identification of Wolbachia from the filarial nematode Mansonella perstans. Mol Biochem Parasitol. 2008;160:123–33 8. DOI: 10.1016/j.molbiopara.2008.04.012

Rao RU, Atkinson LJ, Ramzy RMR, Helmy H, Farid HA, Bockarie MJ, et al. A real-time PCR-based assay for detection of Wuchereria bancrofti DNA in blood and mosquitoes. Am J Trop Med Hyg. 36 2006;74:826–32.
